# Puerperal Fever

**Published:** 1847-12

**Authors:** 

**Affiliations:** Ohio


### SELECTIONS

FROM

### AMERICAN AND FOREIGN JOURNALS.

---

*Puerperal Fever.* By Dr. Dawson, of Ohio.—During certain seasons, we see that the proportion of females affected is much greater than in others. As a general rule, this happens when typhus, erysipelas, or something of the kind is prevailing. In hospitals, where the atmosphere is always more vitiated than in private practice, the complaint has always been more frequent. It seems to us, from what testimony we have, that it prevails also more frequently, and perhaps with more severity, in malarious districts, or in localities where the atmosphere is always more or less vitiated, than the other. Besides these suggestions, every practitioner who has had much experience is aware that the complaint at times springs up in his practice in such a way that leaves him perfectly destitute of all means to account for its origin, without referring it to the agency of a vitiated condition of the atmosphere. That contagious matter exists in the gaseous form, and that it is as capable of producing its deleterious effects as well in this form as in any other, is a position so evident that it would be a work of supererogation to insist upon its correctness in this place.

Besides being communicated through the medium of a tainted atmosphere, there are strong reasons for believing that the erysipelatous inflammation of puerperal females owes its origin not unfrequently to a process something like direct inoculation. How otherwise are we to explain the origin of those cases that are exclusively confined to the practice of a single accoucheur, when little or nothing is known of the disease by other individuals practicing in the same department? Mr. Robertson, of Manchester, states that from the 3d of December, 1830, to the 4th of January, 1831, a midwife attended thirty patients for a public charity; sixteen of these were attacked with puerperal fever, and died. He also states that in the same month, three hundred and eighty women were delivered by other midwives for the same institution, but none of the three hundred and eighty suffered in the smallest degree. Facts of this kind might be largely multiplied, furnishing irresistible evidence that something more than mere contagion, operating through the medium of a tainted atmosphere, must be invoked to assist in accounting for the origin of the malady.

It is a fact well settled by numerous observations, that disease can be excited in a system decidedly healthy, by placing any portion of it, from which the cuticle has been removed, in contact with certain substances in a state of decomposition.

The virus of smallpox, we know, when introduced into the stomach, is incapable of exciting its own peculiar set of morbid phenomena. In this situation, it is very probable that it is digested. Brought, nevertheless, in contact with a small puncture of the lancet made on any part of the body, it soon reaches the circulation, and produces its effects by exciting a series of changes or such a transposition in the elements of the blood as is similar in all respects to the one by which the changes were first excited.

Several hundred cases are known in which serious disease and death have been brought about by the use of a certain kind of sausages. In the manufacture of this kind of food, blood, liver, brains, milk and bread are mixed together, with salt and spices, and then put into intestines, and boiled and smoked. It occasionally happens, when the salt and spices are deficient, and the article in other respects is imperfect, that a peculiar kind of decomposition commences at the center of the sausage, and without any appreciable escape of gas, they become paler in color, soft and greasy, and are found to be in a state of decomposition. The disease which follows the use of this poisonous kind of food is of a very singular character: there is a gradual wasting of all the organs and tissues of the body, and the patient, after becoming emaciated, dries into a complete mummy, and dies; the carcass is stiff and but slightly subject to putrefaction. For some time it was supposed that this kind of food contained a certain kind of poison, to which its effect should be ascribed. Chemists, however, have been unable to detect in it anything like an agent of this character. It contains, we are told, free lactic acid or lactate of ammonia, universal products of animal and vegetable decomposition. As a consequence, the deleterious effects are now ascribed to the condition or state in which the elements are found to exist. Being in a state of transformation, they impart their own peculiar kind of motion to the coats of the stomach, and through the tissues of these organs it is communicated to the blood.

Long since, the profession have been made familiar with the fact that bodies in anatomical rooms frequently pass into a certain state which, is capable of imparting itself to the living body through the medium of the slightest cuts on the hand, and of producing the most serious consequences.

Colin made a number of experiments which clearly prove that muscle, urine, cheese, cerebral substances, etc., in a state of putrefaction, communicate their own state of decomposition to substances much less prone to change of composition than human blood. Placed in contact with a solution of sugar, they cause its putrefaction, or the transposition of its elements into carbonic acid and alcohol.

It is further known that putrefying blood or flesh, placed upon a fresh wound, causes vomiting, disease and death.

How or in what manner all such phenomena are to be explained is one of the questions well calculated to puzzle the most ingenious. By invoking the aid of a certain law, that holds true in several departments of physics, we may approximate possibly towards an explanation. The law to which we allude is, that "a molecule set in motion by any power can impart its own motion to another molecule with which it may be in contact;" and hence the mere contact of any susceptible portion of the system with certain animal matters in a state of decomposition is, if this law holds true, sufficient to excite the same kind of action among the constituents of the blood.

The human organism in a certain sense may be regarded as a chemical compound, made up of elements arranged together according to the laws governing chemical equivalents, and being besides under the influence of the vital force.

Like all other compounds, but not perhaps to the same extent, it is liable to be acted on by certain agents possessing a noxious influence. The blood (which by its nature and constitution is one of the most complex of all the matters of this compound, from the fact that, although not properly speaking an organ, it is nevertheless the sum of all the organs in a state of solution,) has been found to offer the most feeble resistance to exterior influences of any other part or constituent of the organism. This is seen in the function of hematosis, in the effects communicated by the bite of serpents and the sting of insects.

Having founded these facts on some of the various modes in which disease is excited, the mind easily passes to the consideration of the great resemblance between several of them and the manner in which it is probable that erysipelatous puerperal inflammation is produced.

The state of the parts concerned in parturition, as all know, is raw and abraded; the communication, therefore, of noxious agents with the blood is from this circumstance rendered easy. The interior of the uterus, indeed, as Dr. Furgeson justly observes, forms a large wound, presenting a striking analogy to the surface of a stump after amputation. In contact with this surface the hand of the accoucheur, as all know, is frequently brought. If, therefore, he has been engaged in dressing sloughing sores, or has had his hands in contact with animal matter of any kind in a state of decomposition or putrefaction, what is there in the circumstances to prevent disease from being communicated by a process similar to direct inoculation? We all know with what tenacity the smell contracted from putrefying animal matter clings to the fingers, in spite of the most thorough and repeated washings. Well, while the odor remains, there must remain also the matter that produces it. It may be supposed that the quantity of poisonous matter here is too small; but we have instances in the inoculated smallpox, in the vaccine disease, in hydrophobia, in the viper bite, and in the scratches and punctures of the dissecting room, of the minute quantity of an animal poison sufficient to corrupt the whole mass of the blood, and fill the body with loathsome disease.

The changes which accrue to the organism from contact with the different kinds of virus to which we have alluded, we do not suppose, are all identical in their character. Different kinds of virus, it may be presumed, give rise to different kinds of morbid action, and the same substance in different states of decomposition, we have no doubt, is capable of exciting different grades of diseased action. All the morbid changes which have been excited by bodies which themselves are in a state of change, as far as we can see, might be generally termed putrefaction; for whether taken in its popular or technical acceptation, this term signifies nothing more than a natural process by which animal and vegetable matters are disorganized, or a disturbance of the equilibrium between the chemical and vital forces by which the former holds the predominant influence. The term fermentation, which signifies an interstitial movement in a body, whence result certain substances that did not previously exist in it, may express a process that has a still closes analogy to the one under consideration.

Upon these views, however, we do not design to insist, further than merely to suggest them for consideration, believing, as we do, that in relation to the intricate subject before us, they are the most plausible that can be proposed.—*Proceedings of Ohio Medical Convention.*

